# Supplementary material for: Recruiters' perspectives of recruiting women during pregnancy and childbirth to clinical trials: A qualitative evidence synthesis
Source: PLoS One. 2020 Jun 19;15(6):e0234783. doi: 10.1371/journal.pone.0234783 (PMC7304625; doi:10.1371/journal.pone.0234783)
Supplement: S2 Table — (DOCX) [file pone.0234783.s002.docx]

## S2 Table. Critical Appraisal Skills Program (quality assessment of included studies)

Adapted by Ames *et al.,* (2017)* *Ames HMR, Glenton C, Lewin S. (2017) Parents’ and informal caregivers’ views and experiences of communication about routine childhood vaccination: a synthesis of qualitative evidence. Cochrane Database of Systematic Reviews, Issue 2. DOI: 10.1002/14651858.CD011787.pub2*

| Study | Assessor | 1. Are the setting(s) and context described  adequately? | Comment | 2. Is the sampling strategy described, and is this appropriate? | Comment | 3. Is the data collection strategy described and justiﬁed? | Comment | 4. Is the data analysis described, and is this appropriate? | Comment | 5. Are the claims made/ﬁndings supported by sufﬁcient evidence? | Comment | 6. Is there evidence of reﬂexivity? | Comment | 7. Does the study demonstrate sensitivity to ethical concerns? | Comment | 8. . Any other concerns? | Overall Assessment of quality |
| --- | --- | --- | --- | --- | --- | --- | --- | --- | --- | --- | --- | --- | --- | --- | --- | --- | --- |
| Chhoa *et al.,* (2017) | VH | √ | Pg. 2&3 | √ | Pg. 2 | √ | Pg. 2 | √ | Pg. 2 | √ | Pg. 3-8 | No |  | √ | Pg.10 | No | Minor concern due to reflexivity |
|  | LB | √ | Pg.2 | √ | Pg. 2 | √ | Interview schedule | √ | Data analysis | √ | In results | No |  | √ | From REC | No |  |
| Hallowell *et al.,* (2016) | VH | √ | Pg. 1 & 2 | √ | Pg. 4 & 5 | √ | Pg. 4 | √ | Pg. 4 | √ | Pg. 5-8 | No |  | √ | Pg. 4 | No | Minor concerns due to reflexivity |
|  | LB | √ | In background | √ | In recruitment | √ | Telephone interview | √ | Constant comparative | √ | In results | No |  | √ | obtained | No |  |
| Lawton *et al*., (2016) | VH | √ | Pg.2 | √ | Pg. 3-4 | √ | Pg. 4 | √ | Pg. 4-5 | √ | Pg. 5-10 | No |  | √ | Pg. 5 | No | Minor concerns due to reflexivity |
|  | LB | √ | In background | √ | Appropriate – few detail | √ | Brief description | √ | Brief but adequate | √ | narrative | No |  | √ | approval | No |  |
| Stuart *et al.,* (2015) | VH | √ | Pg. 780 | √ | Pg.781 | √ | Pg.781 | √ | Pg.781 | √ | Pg.781-784 | No |  | √ | Pg. 781 | No | Minor concerns due to reflexivity |
|  | LB | √ | In background | √ | In participants | √ | Described not justified | √ | Thematic analysis | √ | Raw data | No |  | √ | evident | No |  |
| van der Zande *et al.,* 2019 | VH | √ | Pg.2 | √ | Pg.3 | √ | Pg.3-4 | √ | Pg. 4 | √ | Pg. 6 | No |  | √ | Pg. 3 | No | Minor concerns due to reflexivity |
|  | LB | √ | In background | √ |  | √ | In study design | √ | Data analysis | √ | Yes | No |  | √ | Framework mentioned | No |  |
