# Supplementary material for: Recruiters' perspectives of recruiting women during pregnancy and childbirth to clinical trials: A qualitative evidence synthesis
Source: PLoS One. 2020 Jun 19;15(6):e0234783. doi: 10.1371/journal.pone.0234783 (PMC7304625; doi:10.1371/journal.pone.0234783)
Supplement: S3 Table — (DOCX) [file pone.0234783.s003.docx]

### S3 Table. Theme Matrix (mapping studies to themes)

|  | | | Studies | | | | |
| --- | --- | --- | --- | --- | --- | --- | --- |
| Themes | Sub-themes | Codes | Chhoa *et al.,* 2017 | Hallowell *et al., 2016* | Lawton *et al., 2016* | Stuart *et al., 2015* | van der  Zande *et al., 2019* |
| 1.Recruitment through the clinician lens | 1.1 Clinical care is the priority | Prioritising clinical care over recruitment |  | X |  | X |  |
|  |  | Challenges for clinician recruiters – Busy/ under-staffed/ pressure/ burdened | X | X |  | X |  |
|  | 1.2 Recruiter’s perception of pregnant women in clinical trials | Pregnant women are vulnerable/fearful | X |  | X |  | X |
|  |  | Perception of women’s motivation/ disinterest | X |  |  | X | X |
|  |  | Perception of women’s mental capacity | X |  | X |  | X |
|  |  | Perception of women’s reading capacity |  |  | X | X |  |
|  |  | Burden of participation on women |  |  |  |  | X |
| 2. The recruiter’s judgement on acceptability | 2.1 Acceptability of the trial | Questioning the research (aim/goal) | X | X |  | X | X |
|  |  | Value/worthiness of trial |  | X | X | X | X |
|  |  | Trial undermines practice |  |  |  | X |  |
|  |  | Recruitment methods and processes (adopting protocol) |  |  | X | X |  |
|  |  | Optimism (incl. therapeutic optimism) |  | X | X |  |  |
|  | 2.2 Acceptability of the intervention | Intervention meets a clinical need |  | X |  |  |  |
|  |  | Intervention is potentially beneficial for clinicians and organisations of care |  | X |  |  |  |
|  |  | Intervention is suboptimal to existing care |  |  |  | X |  |
|  |  | Perception of risk attached to intervention | X | X |  | X | X |
|  |  | Familiarity with intervention reduces risk perception |  | X |  |  |  |
|  |  | Recruiting and delivering intervention – complement/clash | X | X |  |  |  |
| 3. From protocol to recruiter’s lived experience | 3.1 Recruiter as gatekeeper | Recruiters are also clinical gatekeepers | X | X | X | X | X |
|  |  | Protective of women and foetus |  |  | X |  | X |
|  |  | Midwife as double gatekeeper | X |  |  | X |  |
|  |  | Gatekeeper for trial (choosing the ‘right’ participant) |  |  |  | X | X |
|  |  | Directive steering of women’s decision making |  |  |  | X | X |
|  |  | Ad hoc approach to recruitment protocol |  | X |  |  | X |
|  | 3.2 Recruitment encounters | Clinicians are best place/vital for recruitment | X | X |  |  |  |
|  |  | Established rapport with women | X |  |  | X | X |
|  |  | Own work practices threatened by trial/recruitment |  |  |  | X |  |
|  |  | More than one encounter | X | X |  |  |  |
|  |  | More than one recruiter | X | X |  |  |  |
|  |  | Verbal/written communication | X |  | X |  |  |
|  |  | Follow up/exit encounter | X |  | X |  |  |
| 4. Framing recruitment in context | 4.1 Situational context | Emergency/time-critical trials – lack of time | X | X | X |  |  |
|  |  | Adapted communication (giving the ‘headlines’) | X |  | X |  |  |
|  |  | Recruitment & consent become fluid | X |  | X |  | X |
|  |  | Question of & proof of informed consent | X |  | X |  | X |
|  |  | Legal awareness | X |  |  |  | X |
|  | 4.2 Research knowledge and understanding of the trial | Methodological awareness | X |  |  | X | X |
|  |  | Knowledge of the trial/recruitment process |  |  |  | X | X |
|  |  | Research culture |  |  |  | X |  |
|  |  | Equipoise |  | X |  | X |  |
|  |  | Sense of accomplishment from recruitment success |  | X | X |  |  |
|  |  | Selling the trial |  |  | X |  |  |
|  |  | Recruiters want to be trained (initially & ongoing) | X | X |  | X |  |
