# Supplementary material for: Recruiters' perspectives of recruiting women during pregnancy and childbirth to clinical trials: A qualitative evidence synthesis
Source: PLoS One. 2020 Jun 19;15(6):e0234783. doi: 10.1371/journal.pone.0234783 (PMC7304625; doi:10.1371/journal.pone.0234783)
Supplement: S4 Table — (DOCX) [file pone.0234783.s004.docx]

## S4 Table. CERQual Evidence Profile (Lewin *et al.,* 2018b)

|  | Summary of review finding | Studies contributing to review finding | Methodological limitations | Coherence | Adequacy | Relevance | *CERQual assessment of confidence in the evidence* | *Explanation for CERQual assessment* |
| --- | --- | --- | --- | --- | --- | --- | --- | --- |
| **Theme 1: Recruitment through the lens of a clinician** | | | | | | | |  |
|  | 1.1 Clinical care is the priority |  |  |  |  |  |  |  |
| 1 | Recruiters in the maternity care setting were predominately clinicians. Primarily identifying as clinicians, recruiters prioritised clinical care over recruitment responsibilities. | 31,32,33,34,35 | **Minor** methodological limitations (reflexivity was not addressed in the five studies) | **Moderate** concerns about coherence (some concerns about the fit between the data from primary studies) | **Minor** concerns about adequacy (four studies offered moderately rich data) | **No concerns** regarding relevance | **Moderate confidence** | Minor concerns regarding methodological limitations and moderate concerns regarding coherence (studies 2 & 3 based on same cohort) |
| 2 | The duality of the clinician/recruiter role meant that recruiters were already busy juggling a high clinical workload, which allowed little time for the task of recruitment. In contrast, recruiters employed purely in a research capacity, experienced recruitment as unhurried and complementary to the women’s care experience. | 31,32,34 | **Minor** methodological limitations (reflexivity was not addressed in the five studies) | **No concerns** about coherence | **Minor** concerns about adequacy (three studies together offered moderately rich data) | **No concerns** regarding relevance | **High confidence** | Minor concerns regarding methodological limitations. |
|  | 1. 2 Recruiters perception of pregnant women in clinical trial | | |  |  |  |  |  |
| 3 | Recruiters made judgements, and assumptions, about pregnant woman’s mental capacity and their ability to comprehend what trial participation involved. Recruiters regarded pregnant women as vulnerable and believed they needed to be protected from research. | 31,33,34,35 | **Minor** methodological limitations (reflexivity was not addressed in the five studies) | **No concerns** about coherence | **Minor** concerns about adequacy (four studies together offered moderately rich data) | **No concerns** regarding relevance | **High confidence** | Minor concerns regarding methodological limitations and adequacy of data. |
| 4 | Paternalism was evident in the recruiter assumption of knowledge regarding how receptive pregnant women would be towards the trial, and also in their use of language regarding potential participants (inference of ownership). | 34,35 | **Minor** methodological limitations (reflexivity was not addressed in the five studies) | **Moderate** concerns about coherence (some concerns about the fit between the data from primary studies) | **Moderate** concerns about adequacy (only two studies offered rich data) | **No concerns** regarding relevance | **Moderate** | Minor concerns regarding methodological limitations, moderate concerns regarding coherence and adequacy of data. |
|  | **Theme 2: The recruiter’s judgement of acceptability** | | | | | | |  |
|  | 2.1 Acceptability of the trial | | | | | | |  |
| 5 | The clinical relevance and aim of the trial were valued by recruiters. Concordance between their views and the research goals facilitated the recruiter’s willingness to engage in recruitment, while discordance dissuaded recruitment. | 32,33,34,35 | **Minor** methodological limitations (reflexivity was not addressed in the five studies) | **Moderate** concerns about coherence (some concerns about the fit between the data from primary studies) | **Minor** concerns about adequacy (four studies together offered moderately rich data) | **No concerns** regarding relevance | **Moderate confidence** | Minor concerns regarding methodological limitations and adequacy of data, and moderate concerns regarding relevance (studies 2 & 3 same cohort). |
| 6 | It was important to recruiters that the information and recruitment pathways of the trial (protocols) were both practical, and an efficient use of the resources available | 33,34 | **Minor** methodological limitations (reflexivity was not addressed in the five studies) | **No concerns** about coherence | **Moderate** concerns about adequacy (moderately rich data from two studies) | **No concerns** regarding relevance | **Moderate confidence** | Minor concerns regarding methodological limitations and moderate concerns regarding adequacy of data. |
|  | 2.2 Acceptability of intervention | | |  |  |  |  |  |
| 7 | Recruiters formed an opinion around the acceptability of the trial intervention based on its utility, potential benefits for stakeholders, and potential to ultimately improve the current standard of care. | 32,33,34,35 | **Minor** methodological limitations (reflexivity was not addressed in the five studies) | **No or very minor** concerns about coherence | **Minor** concerns about adequacy (four studies offered moderately rich data | **No concerns** regarding relevance | **High confidence** | Very minor concerns regarding coherence and minor concerns regarding methodological limitations and adequacy of data (studies 2 & 3 same cohort) |
| 8 | Recruiters expressed optimism and hope for a successful trial outcome.  Their emotional engagement triggered a cyclical process where the recruiters ‘buy in’ to the trial generated recruitment, leading to a sense of achievement, which in turn provided positive reinforcement for their efforts and generated further recruitment. However, the cycle could also be reversed when declining recruitment rates lead to disengagement. | 32,33,34 | **Minor** methodological limitations (reflexivity was not addressed in the five studies) | **Moderate** concerns about coherence (some concerns about the fit between the data from primary studies) | **Moderate** concerns about adequacy (data from two studies) | **No concerns** regarding relevance | **Moderate confidence** | Minor concerns regarding methodological limitations, moderate concerns regarding coherence and adequacy of data (studies 2 & 3 same cohort). |
| 9 | Recruiters had strong ties with established clinical practice and were uncomfortable recruiting for a trial that shifted away from their routine. | 34,35 | **Minor** methodological limitations (reflexivity was not addressed in the five studies) | **No or very minor** concerns about coherence | **Serious** concerns about adequacy (data only from two studies, offered thin data) | **No concerns** regarding relevance | **Low confidence** | Very minor concerns regarding coherence, minor concerns with methodological limitations, and serious concerns regarding adequacy of data. |
| 10 | The recruiter’s perception of risk (associated with the intervention) was fundamental in their judgement of acceptability, and therefore a key determinant in their willingness to engage in recruitment. Recruiters were more comfortable recruiting to a trial they considered to be low risk. | 31,32,34,35 | **Minor** methodological limitations (reflexivity was not addressed in the five studies) | **Minor** concerns about coherence (some concerns about the fit between the data from primary studies) | **Minor** concerns about adequacy (four studies together offered rich data) | **No concerns** regarding relevance | **High confidence** | Minor concerns regarding methodological limitations, coherence, and adequacy of data. |
|  | **Theme 3: From protocol to lived experience** | | | | | | |  |
|  | 3.1 Recruiters as gatekeepers | | | | | | |  |
| 11 | Recruiters were also gatekeepers to potential participants and assumed the role of a protective advocate of women. Recruiters were paternalistic in the role and were seen withholding trial information and steering women towards decision making which was aligned with their own views. The role of gatekeeping extended to protecting the trial itself. | 31,32,33,34,35 | **Minor** methodological limitations (reflexivity was not addressed in the five studies) | **Minor** concerns about coherence (some concerns about the fit between the data from primary studies) | **Minor** concerns about adequacy (five studies offered moderately rich data) | **No concerns** regarding relevance | **High confidence** | Minor concerns regarding methodological limitations, coherence, and adequacy of data (studies 2 & 3 same cohort) |
| 12 | An additional layer of gatekeeping existed between midwife recruiters and recruiters from other professional backgrounds. In what was apparently a culturally appointed hierarchy, clinical recruiters/gatekeepers sought permission from the midwife recruiter/gatekeeper to approach a potential participant | 31,34 | **Minor** methodological limitations (reflexivity was not addressed in the five studies) | **No or very minor** concerns about coherence | **Serious** concerns about adequacy (only two studies, both offered thin data) | **No concerns** regarding relevance | **Low confidence** | No or very minor concerns regarding coherence, minor concerns with methodological limitations, and serious concerns with adequacy of data. |
| 3.2 Recruitment encounters | | | | | | | | |
| 13 | Recruitment frequently involved a team approach, with often more than one recruiter over several encounters. Recruiters often engaged in an ‘exit’ encounter, post-trial, which appeared to bring closure to the trial recruitment process for both women and recruiters. | 31,32,33 | **Minor** methodological limitations (reflexivity was not addressed in the five studies) | **Minor** concerns about coherence (some concerns about the fit between the data from primary studies) | **Moderate** concerns about adequacy (studies offered thin data) | **No concerns** regarding relevance | **Moderate confidence** | Minor concerns regarding methodological limitations and coherence. Moderate concerns regarding adequacy of data (studies 2 & 3 same cohort). |
| 14 | There was no consensus reached amongst recruiters on the best method and at what stage trial information should be communicated. | 31,33 | **Minor** methodological limitations (reflexivity was not addressed in the five studies) | **Minor** concerns about coherence (some concerns about the fit between the data from primary studies) | **Moderate** concerns about adequacy (only two studies, offered thin data) | **No concerns** regarding relevance | **Moderate confidence** | Minor concerns regarding methodological limitations and coherence. Moderate concerns regarding adequacy of data |
|  | **Theme 4: Framing recruitment in context** | | | | | | |  |
|  | 4.1 The situational context | | | | | | |  |
| 15 | Recruiting pregnant women during an emergency or in a time-critical situation challenged recruiters to communicate effectively in a highly pressured time constrained environment. Recruiters were not comfortable with the task of obtaining informed consent in this environment | 31,33 | **Minor** methodological limitations (reflexivity was not addressed in the five studies) | **Minor** concerns about coherence (some concerns about the fit between the data from primary studies) | **Minor** concerns about adequacy (two studies both offered rich data) | **No concerns** regarding relevance | **Moderate confidence** | Minor concerns regarding methodological limitations, coherence and adequacy of data. |
| 4.2 Research knowledge and understanding of the trial | | | | | | | | |
| 16 | Training in methodological aspects of the trial and recruitment protocols provides recruiters with the knowledge and confidence to perform their recruitment task well. Recruiters recognised the need for regular structured multidisciplinary recruitment training, both initially, and throughout life of trial | 31,32,34,35 | **Minor** methodological limitations (reflexivity was not addressed in the five studies) | **No or very minor** concerns about coherence | **Minor** concerns about adequacy (four studies together offered moderately rich data) | **No concerns** regarding relevance | **High confidence** | No or very minor concerns regarding coherence, minor concerns with methodological limitations and adequacy of data. |
