# Supplementary material for: Recruiters' perspectives of recruiting women during pregnancy and childbirth to clinical trials: A qualitative evidence synthesis
Source: PLoS One. 2020 Jun 19;15(6):e0234783. doi: 10.1371/journal.pone.0234783 (PMC7304625; doi:10.1371/journal.pone.0234783)
Supplement: S5 Table — (DOCX) [file pone.0234783.s005.docx]

## S5 Table. Exemplars of supporting data

| Theme | Supporting Data |
| --- | --- |
| Theme 1: Recruitment through a clinician’s lens | |
| 1.1 Clinical care is a priority | *‘… in our team we're very short staffed, we are trying their best to recruit people, but it's quite hard…if the stafﬁng situation was better, and there wasn't as much stress there might be more referrals.’ (Midwife, Stuart et al., 2015)*  *“I do find that it takes an awful long time, it’s not news to spend an hour or more talking to them. I have spent three hours with a family, just because they’ve got so many questions and there’s so much else going on” (Neonatal Nurse, Chhoa et al, 2017)*  *“and it’s not done in a hurry like oral (*consent)*, loads of loads of opportunity to just go and to chat and to talk about stabilisation and to talk about the relationship between mother and baby.” (Research Midwife, Chhoa et al 2017)* |
| 1.2 Recruiter’s perception of pregnant women in clinical trials | *“There’s a simplified version which is much more straightforward, and I think this is probably they level you should be aiming for when someone’s just had a baby. I don’t mean that in a condescending way, I just mean they are exhausted, and they’ve often been up for 2 days so giving them too much information is unfair really.” (Doctor, Lawton et al, 2016)*  *“Well I think generally in any study you have to remind people and make sure that they fully understand, but also the specifics because if the woman is in labour or delivery is threatened early I still feel that they are not in the full frame of mind to grasp everything that you’re saying” (Neonatal registrar, Chhoa et al 2017)*  *“Assuming that women may function differently during their pregnancy, also psychologically, you don’t know if that does not influence their decision-making surrounding research participation” Gynaecologist, van der Zande et al 2019)*  *“they (*pregnant women) *ﬁnd groups really difﬁcult because a lot of my ladies aren't very articulate, a lot can't read.” (Midwife, Stuart et al, 2015)*  *“Because the women, they don’t, in my opinion, have the ability to read the information themselves and retain everything.” Midwife, Lawton et al., 2016)*  *“it appeared that the main reason why stakeholders are reluctant to include pregnant women in clinical research stems from this felt need to protect the woman and her foetus” (Author, van der Zande 2019)*  *‘There were two ladies that just ﬁtted the criteria. We didn't pass the details on as we know the women wouldn't come to group.’ Midwife, Stuart et al 2015)* |
| Theme 2: Recruiter’s judgement of acceptability | |
| 2.1 Acceptability of the trial | *“Yeah, it’s definitely a good, good idea, and the thinking about behind it is fairly reasonable as well, in terms of using GTN to relax the uterus to deliver the placenta. Yeah, so I think it’s a really good trial.” (Doctor, Hallowell et al, 2016)*  *“…at times it may happen that healthcare professionals are hesitant to include their patients… when the professionals for example… believe that the study itself is not relevant.” (Author, van der Zande et al., 2019)*  *“…in the sense that people recognise the drug, so instantly can start to hypothesise what the rationale is for the trial, without reading anything.” Doctor, Hallowell et al., 2016)*  *‘We seem to be researching the same issues about giving women more support then they'll do better, but it seems it's always targeted at speciﬁc groups that don't tend to want to take part anyway. I think it would be better to spend the resources in a general population” (Midwife, Stuart et al, 2015)*  *“I think it was quite difﬁcult because the criteria were so strict, and, so they ﬁt one part of the criteria, but then failed to ﬁt on another” (Midwife, Stuart et al, 2015)*  *“‘If you stop to tell everybody about this trial, but only a couple of percent will have it (a retained placenta), it’s gonna waste so many resources.” (Dr J, Lawton et al, 2016)*  “… (the trial represented) *an intrusion into their domain which could be perceived as a threat to their own way of working” (Author, Stuart et al, 2015)* |
| 2.2 Acceptability of the intervention | *“the potential participants really want something, and you have something to offer” (Doctor, van der Zande et al., 2019)*  *“And there are so many benefits I think the midwives are keen to do it…. And especially for the future, if it does work, being able to use in birth centres and community settings.” (Research midwife, Hallowell et al., 2016)*  *“But to be honest, I think the only incentive that I, or any midwife, would need is the possibility that it might work.” (Midwife, Hallowell et al., 2016)*  *“I think the impact will be a positive impact” (Midwife, Stuart et al., 2015)*  *“A lack of success…, which resulted in staff emotionally and (potentially) physically disengaging from the trial, as they began to perceive the research as conflicting with their duties of care… research staff also suggested that declining TO (and recruitment rates) and a negative shift in levels of uncertainty at an individual/site level could be reversed by a run of successful outcomes” (Author, Hallowell et al., 2016)*  *“Moreover, the internal exam was, contrarily to the practice at the AMC, not part of standard care and thus became an extra invasive procedure which was considered to be a barrier for both pregnant women and healthcare professionals.” (Author, van der Zande et al., 2019)*  *“It could be dangerous, ….” (Midwife, Stuart et al., 2015)*  *“Because there’s not any risks to what we’re doing, then it would be a shame to miss them if it’s something that they would have liked to have been involved in.” (Neonatal Nurse, Chhoa et al., 2017)*  *“because staff were familiar with GTN and perceived it as a relatively risk-free intervention” (Author, Hallowell et al., 2016)*  *“While they did not know what the outcome of the trial would be, the fact that they had used GTN before to beneficial effect influenced their views of the trial and their role in trial delivery…and perceived it as a relatively risk-free intervention” (Author, Hallowell et al., 2016)*  *‘Cause we’ve had quite a few PPHs. And obviously, we don’t know if its linked or not. But it’s put a lot of the midwives, and doctors [off]*(recruiting) *… (Research Midwife, Hallowell et al., 2016)* |
| Theme 3: From Protocol to recruiter’s lived experience | |
| 3.1 Recruiter’s as gatekeepers | *“See the big thing really for us has been the hospital staff being on board…They’ve been really good…* *… cause without them on board we’d not get anywhere with it. (Research midwife, Hallowell et al., 2016)*  *“It still has to come from us otherwise you'd have to have the other [gFNP] midwife coming in right at the start, midwives would have to identify women for a roll-out.” (Midwife, Stuart et al., 2015)*  *“I did ask the midwives if it was reasonable to make an approach” (Consultant neonatologist, Chhoa et al., 2017)*  *“Sometimes ignorance is bliss.” (Midwife, Lawton et al., 2016)*  *“at times it may happen that healthcare professionals are hesitant to include their patients and make a conscious choice to “counsel negatively”. (Author, van der Zande et al., 2019)*  *“Of course, in clinical practice midwives can exercise their judgement about which of the potentially eligible women to refer to gFNP whereas for the trial they were being asked to pass on all relevant names, which is an important difference.” (Author, Stuart et al., 2015)* |
| 3.2 Recruitment encounters | *“If we’ve approached a woman during the antenatal counselling giving them a leaflet, then that will then be backed up by a research nurse going to them and taking their consent the following day. And I think that system works really, really well.” (Consultant Neonatologist, Hallowell et al., 2016)*  *“clinicians often reported meeting with women and their partners and explaining the study at least several times before they asked the woman to make a decision about participation” (Author, Chhoa et al., 2017)*  *“oral assent may help a woman in labour make a more informed decision as the focus is not on signing a piece of paper, but rather on the conversation.” (Author expressing Research Midwife’s view, Chhoa et al., 2017)*  *“staff were reluctant to consider other approaches, a position which did not change when individuals were told, during their interviews, that women had expressed a wish for earlier information giving.” (Author, Lawton et al., 2016)*  *“most appropriate person to initially provide information about the study was one of the neonatal staff as part of their antenatal counselling and that this should be embedded in the “normal care of ladies having preterm babies” (Research Midwife, Chhoa et al., 2017)*  *“Staff did, however, highlight the potential benefits of revisiting trial information and offering a post-trial debriefing which, as MW N suggested, would allow women to better understand ‘what was happening because their head’s a bit more together” (Author, Lawton et al., 2016)*  *“I’ve often done that social call afterwards” (Research midwife, Chhoa et al., 2017)* |
| Theme 4: Framing recruitment in context | |
| 4.1 The situational context | *“It’s an interesting environment to consent patients for a clinical trial. Because it’s quite different to a sort of, you know, sit down clinic, have a think about something, then write to me if you’re interested” (Doctor, Lawton et al., 2016)*  *“…some have seconds to make the decision and some have ten minutes to make the decision, but I think there’s a massive difference in that.” (Research Nurse, Chhoa et al., 2017)*  *“… if it’s the headlines then they get their head around a few salient points, then they’re more likely to take that on board then be able to say yes or no, rather than ‘actually there’s too much, I can’t actually think about that now” (Consultant Neonatologist, Chhoa et al., 2017)*  *“staff discussed how they had tended to simplify and give ‘the minimum of information’ (Dr J) they thought was needed to gain informed consent” (Author, Lawton et al., 2016)*  *“… and would she just agree to anything at that point because she’d rather just get on with having her baby than talking to you” (Neonatal Registrar, Chhoa et al., 2017)*  *“is this a water-tight document? Have you mentioned everything possible that you need to mention? Is it comprehensive, detailed enough to evidence the consent process?” (Consultant neonatologist, Chhoa et al., 2017* |
| 4.2 Research knowledge & understanding of the trial | *“We want the data that we get for the trial to be generalisable because otherwise you’re going to end up with different patient groups and your trial is not going to be valid, your outcome’s not going to be valid” (Con. Neonatologist, Chhoa et al., 2017)*  *“If there is a study where you think ‘I’m not sure what I’m doing here’, it is definitely a reason to counsel in the other direction. You try to counsel objectively, but we all know it is directive.” (Midwife, van der Zande et al., 2019)*  *“…our team leader didn’t go through it, she just said there's a pack in everybody's drawer to refer people, I spoke to some of the other midwives… they said exactly the same thing, it was just in the drawer… under a pile of notes. Which is awful, because I think probably, we missed a lot of people not knowing.” (Midwife, Stuart et al., 2015)*  *“Providing training throughout the lifetime of a trial that allows staff to reflect upon how their TO* [Therapeutic Optimism] *about the trial is sustained, maintained and reinforced by their trial and other experiences might similarly be helpful and ensure that TO* [Therapeutic Optimism] *does not bias recruitment rates.” (Author, Hallowell et al., 2016)*  *“The importance of regular multidisciplinary training for offering participation in the trial was also commented upon by clinicians” (Author, Chhoa et al., 2017)*  *“It is also essential that midwives have sufﬁcient understanding of the various approaches to participant recruitment and this requires continuing attention in midwifery education.” (Author, Stuart et al., 2015)*  *“One difficulty raised by some clinicians was the rotation of registrars, and the need to ensure new staff members were trained in offering consent was highlighted.” (Author, Chhoa et al., 2017)*  *“providing staff with training about the conceptual underpinning of trial delivery — clinical equipoise — and getting them to acknowledge their lack of individual equipoise or trial preferences facilitates trial delivery by enabling them to take a more objective stance towards trial recruitment and delivery” (Author, Hallowell et al., 2016)* |

Key

Primary study participant quote

Primary study author
