# Supplementary material for: Recruiters' perspectives of recruiting women during pregnancy and childbirth to clinical trials: A qualitative evidence synthesis
Source: PLoS One. 2020 Jun 19;15(6):e0234783. doi: 10.1371/journal.pone.0234783 (PMC7304625; doi:10.1371/journal.pone.0234783)
Supplement: S1 Appendix — (DOCX) [file pone.0234783.s006.docx]

## S1 Appendix. SPIDER Table of terms and search strategy

| SPIDER Table of Terms |  |  |
| --- | --- | --- |
| S - Sample | Recruiters  *(clinical & non-clinical)* | |
| PI - Phenomenon of Interest | Recruitment to clinical trials  *of women during pregnancy & childbirth to clinical trials* | |
| D - Design | Any design including qualitative data  *Interviews, case studies, focus groups, observations etc.* | |
| E – Evaluation | Views of recruiters  *Experiences, perceptions, attitudes, beliefs* | |
| R - Research Type | Qualitative  *All identifiable qualitative data* | |

“In particular, we believe that adapting the logic that originally underpinned the SPIDER tool, so that S and PI are initially combined with “AND” and then, in turn, “AND-ed” with the three methodological terms (namely “D OR E OR R”), might prove a fruitful line of inquiry. This might particularly be the case if the searcher could aim to make the S and PI specific to the indexing language of the database, but, in contrast, to make the D, E, and R sensitive for qualitative research-related terms. Such an approach would be particularly justified, in line with the earlier methodological discussion, where the intention is to sample the most relevant qualitative research articles (as for an interpretive review) rather than to search exhaustively and comprehensively (as for an aggregative review).” Cooke *et al*., (2012)

**Search Strings**

**S** - Recruiter* OR Trialist* OR Researcher* OR Midwives OR Midwife* OR Nurs* OR Clinician* OR Physician* OR Doctor* OR investigator

AND

**PI** - recruit* OR participa* OR enrol* OR accrual OR non-participa* OR nonparticipa* AND Wom*n OR pregnan* OR pre-natal OR prenatal OR ante-natal OR antenatal OR peri-natal OR perinatal OR trimester OR mother OR maternal OR Birth OR childbirth OR child-birth

AND “clinical trials” OR trials

**D** - ethnograph* OR phenomen* OR “grounded theory” OR hermeneutic* OR “case study” OR observation OR “focus group* OR Interview*

**E** - experience* OR attitude* OR belief* OR perceive OR perception OR opportunit* OR opinion* OR agree* OR accept OR refus* OR decline OR decision OR decide OR factor* OR enable OR Succe* OR fail* OR facilitat* OR barrier* OR obstacle* OR challenge* OR problem* OR issue*

**R** - Qualitative OR “mixed-method” OR “mixed method” OR “multi-method” OR “multi method” OR thematic analysis OR narrative

| *Inclusion* | *Exclusion* |
| --- | --- |
| All qualitative studies such as grounded theory studies, phenomenological studies, narrative studies, ethnographies, case studies, collaborative forms of research, and visual studies | Quantitative studies, commentary articles |
| Studies using qualitative methods for data collection, such as focus groups, face-to-face interviews, observations, arts-based methods or document analysis, and for | All studies not published in English will be excluded (due to limitations in translation resources) |
| Studies using qualitative data analysis such as content analysis, thematic analysis, constant comparison, or other qualitatively inspired analytical approaches | Studies using not using qualitative data analysis methods |
| Mixed methods studies where findings can be identified and extracted from the qualitative research arm separately | Mixed methods studies where qualitative data cannot be identified and extracted separately |
| Studies focusing on the perceptions and experiences of the recruiter when recruiting women during pregnancy & childbirth to clinical trials | Studies focused on women’s experiences for being recruited to clinical trial |
| Studies concerned with clinical trial recruitment | All studies not recruiting to clinical trials (i.e. case studies, cohort studies) |

**Search Filters -** Human, English Language
