# Supplementary material for: Recruiters' perspectives of recruiting women during pregnancy and childbirth to clinical trials: A qualitative evidence synthesis
Source: PLoS One. 2020 Jun 19;15(6):e0234783. doi: 10.1371/journal.pone.0234783 (PMC7304625; doi:10.1371/journal.pone.0234783)
Supplement: Attachment — Submitted filename: PLOS ONE reviews March 2020.docx [file pone.0234783.s007.docx]

Thank you for the opportunity to read this Qualitative Evidence Synthesis investigating recruiter experiences and perceptions of barriers and facilitators to recruitment to randomised trials conducted during pregnancy and childbirth.

I enjoyed reading this systematically presented and well written study. Findings include new interpretations, arguing knowledge and power interact and influence recruitment in this context. The authors suggest findings could have relevance to others of the top 10 areas identified for PRIORITY (specifically how best to ensure inclusion of underrepresented and vulnerable groups and how best to ensure manageable workloads for clinical and research staff who recruit to trials - p 35).

There is a lack of research involvement of women recruited during pregnancy and childbirth and this QES will contribute important insights into recruiter perspectives in an area where knowledge is currently lacking.

This work deserves publication. My comments focus mainly on making more explicit 1) the methods of the QES; 2) clarifying findings.

**Abstract**

1. Suggest avoid ‘underpinned’ and replace with ‘linked’ or other rewording. Describing an ‘overarching theme’ as an ‘underpinning’ comes across as a contradiction.

**Introduction**

2. P4 Final sentence of first paragraph: Do the authors feel that findings could have relevance to trial recruiters in other clinical areas? This is mentioned on p34 second paragraph although not stated explicitly there or here. Perhaps something to comment on more explicitly either here or in the discussion.

**Methods**

3. P15 Methodology of synthesis: The authors have used the ENTREQ Checklist well in presenting this review. Whilst I accept that a thematic synthesis is justified for the study question, the authors have not yet clearly stated the rationale for conducting a QES/using thematic synthesis (as opposed to using other synthesis methodology). See Booth et al. 2016 [18] for clear arguments for why a thematic synthesis is appropriate for this study.

4. Did the line by line coding reported here include all text in the findings and discussion of the papers reviewed – currently the authors refer to ‘all relevant text’ and it is not clear how a decision on relevance was reached. I was not initially clear whether it was simply participant quotes or also concepts proposed by authors in primary studies (first and second order concepts) and this only became clear on reading Table S5.

5. The authors have not yet made completely clear whether primary studies were analysed in a particular order or whether analysis was iterative across all study data.

**Findings**

6. P20 sixteen summary of qualitative findings statements are reported (Table 2) but I was not clear from the analysis how these were derived – were they derived after the themes were identified or were they derived then grouped together to form themes?

7. P17-19 Table 2: Some content assigned to different themes appeared very closely related, so I was not clear how authors arrived at these themes (see above). Please see summary of qualitative findings statements given in the table below for my alternative interpretation of these SQF statements into three themes (1. Clinician first, recruiter second, 2. Recruiter beliefs about trial, participation and women, 3. Impact of context). Apologies if I have misunderstood the analysis process and I accept the authors are more familiar with the raw data, which may account for the themes presented.

8. General point re findings: I was not always clear which were the authors’ interpretations of the data, which were the interpretations of the primary study authors and what were those of the original research participants – although this became clearer reading S5 Table. Is it possible to signpost this in the text? (p8 France et al. 2019 i.e. distinction between first-order constructs, second order constructs and third order constructs: <https://bmcmedresmethodol.biomedcentral.com/articles/10.1186/s12874-019-0670-7> ).

9. P20-21 There is no mention in the text whether views and experiences differed across these professional groups – I was particularly interested to know if there was variation across professions in views expressed under 1.2: ‘These judgements were based on their perceptions that pregnant women lacked the capacity to understand and make decisions around trial information.’ I see from S5 Table that these views are held across professions I was also curious to know whether any patterns of assumption emerged according to professional group but numbers may have been too small.

10. The authors mention that midwives were regarded by fellow professionals as gatekeepers – was the use of paternalistic language e.g. ‘our girls’, ‘my ladies’ limited to midwives or common across all professions?

11. P22 suggest ‘receptive’ to replace ‘receptable’

12. P22 suggest ‘implied’ in place of ‘inferred’.

**Discussion**

13. P31 The authors sum up their key findings as follows, arguing they have discovered an:

‘overarching theme which combines knowledge and power. In other words, our review suggests that when it comes trials recruitment, recruiters believe they know ‘best’ Recruiters often cite their assumed knowledge and wisdom regarding women’s preference, capabilities and personal circumstances alongside their judgements and assumptions in relation to the trial and the trial intervention’. (p31, first paragraph of discussion).

I agree with the suggestion that there is an interaction between (a lack of) knowledge (or perhaps beliefs rather than knowledge) and power, but I wonder whether this summary of the findings does not make this interaction explicit enough? Is perhaps the key issue that recruiters believe they know best from a clinicians’ perspective (when from a trial conduct perspective they may not) and may act in line with their (clinical) beliefs rather than in line with trial protocol, e.g. in exercising their beliefs about which individuals they should approach rather than approaching all eligible women (gatekeeping which has implications for recruitment and potentially generalisability of findings). If that is the authors’ derstanding, I would like to see this argued clearly here. This then also supports the argument for the need to ensure recruiter training (Implications for practice p 34) as these beliefs may be addressed in training that increases recruiter understanding of trial conduct.

14. P31 second paragraph in the Discussion – I was not clear on the key point that is made and illustrated here. Perhaps that recruiters make assumptions that are often over negative which results in people being excluded from trial participation? Important to make a reference to the implications of this for the generalisability of trial findings (referenced in introduction but helpful to reference here). Maybe the authors could make more explicit that the concept of vulnerability for pregnant women is coming under challenge in the literature but this may not have filtered through to clinical practice.

15. P32 Has the paternalistic language used been reported and critiqued elsewhere in the literature given how striking its use is in this setting? I see the reference to a blog from 2018 but I am curious to know of any other critiques.

16. P33 Paragraph beginning ‘The overarching concept of knowledge’ – perhaps add a comment here that there was evidence of recruiters acting ‘with confidence’ but in a way that contradicted trial protocol, i.e. in line with recruiter beliefs rather than inline with deeper understanding of trial aims and processes.

17. P34 re ‘variation in the approach to the recruitment encounter’. Might this variation be necessary and justified given the wide range of trial designs, point on pathway and setting where recruitment can occur?

18. P35 the authors argue findings could have relevance to other key priorities of research identified by the Priority Study. Maybe also comment on the generalisability of findings for recruiting in other clinical specialties. This is touched upon on p34, first full paragraph but could be brought out more.

19. P35 First sentence under Implications should read ‘Interventions such as the QuinteT Recruitment Intervention…

**Conclusion**

20. P36 Felt this summed up key findings really nicely 1) interaction between belief and power 2) gap between study design and lived reality and how to address these by involving recruiters as key stakeholders at the design phase and providing ongoing training during trial delivery.

**General minor points**

Some variation in tense used – occasional switch from past to present. Helpful for reader to keep consistent to past throughout.

Please check use of semi-colon vs colon.

P22 Sentence beginning ‘Whilst recruiters in Stuart’s and Lawton’s studies … please review sentence construction.

Check for use of ‘inferred’ to mean ‘implied’

P32, first full paragraph, second sentence beginning ‘Particularly striking…’ please check grammar, phrasing.

P32 First sentence of second paragraph: perhaps ‘illustrated in the **language** of paternalism’?

**Additional tables**

S2 Columns 5 and 7 need resizing to show all text.

Should the entry for Stuart et al. by LB in column 8 be entered in column 10? (4. Is data analysis described and is this appropriate – comment)

Given that none of these studies were judged to include evidence of reflexivity is this worth drawing attention to in the discussion, as there is clearly a need to improve this aspect of in future studies?

**Possible organisation of SQFs**

| **Clinician first, Recruiter second** | **Recruiter beliefs about trial, participation and women** | **Influence of context** |
| --- | --- | --- |
| 1. Identify as clinicians and prioritise clinical care over research | 5. Impact of concordance / discordance between R views and research goals. | 15. Time-critical situation challenging and uncomfortable for recruiters |
| 2. clinicians have to juggle workload to fit in recruitment as well as clinical work. | 6. info & recruitment pathways must be practical and efficient | 16. Training needed for confidence and knowledge, both initially and throughout the trial |
| 9. strong ties to existing clinical practice & perception of possible threat to routine | 7. acceptability of trial intervention based on utility, benefits, potential to improve current standard of care | 13. Often used a team approach, more than one encounter/recruiter. Exit encounters. |
|  | 10. acceptability judgement based on judgement of risk of intervention | 14. No consensus on optimum method or stage for providing info – perhaps linked to wide range of contexts needing a flexible approach? |
|  | 8. Optimism re positive trial outcome could lead to virtuous cycle but negative views could lead to a negative spiral |  |
|  | **R Judgements/beliefs about women** |  |
|  | 3. R made judgements and assumptions re women’s capacity, regarded as vulnerable and in need of protection |  |
|  | 4. Paternalism expressed in language & in assumptions re how receptive women might be |  |
|  | 11. R as gatekeepers, protective advocate of women, protective of trial |  |
|  | 12. Gatekeeping vis a vis other professionals (midwives) |  |
