# Supplementary material for: Recruiters' perspectives of recruiting women during pregnancy and childbirth to clinical trials: A qualitative evidence synthesis
Source: PLoS One. 2020 Jun 19;15(6):e0234783. doi: 10.1371/journal.pone.0234783 (PMC7304625; doi:10.1371/journal.pone.0234783)
Supplement: Attachment — Submitted filename: Response to Reviewers.docx [file pone.0234783.s008.docx]

National University of Ireland Galway	PLOS One Journal

School of Nursing & Midwifery,	San Francisco,

Aras Moyola,	California,

Upper Newcastle Rd,	United States

Galway,

Ireland

24/04/2020

Reference: PONE-D-20-04175

Dear Dr Mathes,

We thank you and the reviewers for a thorough reading and helpful feedback of our manuscript and for the opportunity to revise and resubmit. We are pleased to submit the improved research manuscript.

“Recruiters’ perspectives of recruiting women during pregnancy and childbirth to clinical trials: a qualitative evidence synthesis”

On the following pages, you will find our response to both the editor and reviewer comments. For ease of reading, we have addressed each reviewer comment individually and presented them in the table below.

On behalf of my co-authors, I thank you for your consideration of this resubmission. We would be glad to respond to any further questions or comments and look forward to hearing from you regarding our submission.

Yours sincerely,

Vivienne Hanrahan

Corresponding author

National University of Ireland Galway

v.hanrahan1@nuigalway.ie

Response to journal requirements

*1. Please provide any updates you might have since the original search was performed in January 2019, or provide a specific rationale for ending your search at that time.*

The search was re-run prior to submission to this journal in February 2020, at this time no further eligible records were identified for inclusion in the QES.

*2. In the methods, please describe how risk of bias was assessed in individual studies (including specification of whether this was done at the study or outcome level, or both, and the specific test employed, such as the I^2 statistic), and how this information was used in any data synthesis.*

As part of the GRADE-CERQual assessment, we considered the methodological limitations of each study. This component of the assessment is analogous to the risk of bias domain used in the GRADE approach for findings from systematic reviews.

*3. In the Methods, please specify any assessment of risk of bias that may affect the cumulative evidence (e.g., publication bias, selective reporting within studies). Please ensure that the specific method of assessment (funnel plot, Egger's test, Begg's test, etc) is mentioned.*

We have described the GRADE-CERQual approach undertaken in the QES in the section titled “Assessment of confidence in the review findings” (Pg.16) and reported the assessment in detail in S4 Table.

*4. We notice that the email address and names included in the supplementary information file may be potentially identifying. Please ensure that the email is shared only with consent; it may be removed at this time if the sender did not consent to the email being published.*

Thank you for bringing this to our attention, the email has been removed from the supplementary files at this time.

Response to reviewer comments

The authors would like to express their thanks and appreciation to the reviewers for the time and consideration given to making these valuable comments and suggestions.

| **REVIEWER #1** | Location |
| --- | --- |
| **1. ENTREQ is a generic reporting guideline for a range of different qualitative evidence synthesis types. The language used in places (such as the abstract) inappropriately elevates this generic QES reporting guideline to the level of a methods manual (which it is not). See for example the abstract 'our synthesis was guided by the ENTREQ statement'. Suggest consider rewriting along the following lines: 'The synthesis was undertaken using Thomas and Harden's three stage thematic synthesis method, and reported following the ENTREQ reporting guideline for generic qualitative evidence syntheses.'** |  |
| Thank you for highlighting this important oversight, the manuscript has been amended accordingly.  *Revised text:*  *“*The synthesis was undertaken using Thomas and Harden’s three stage thematic synthesis method and reported following the ENTREQ guideline.” | Pg. 2  Ln. 41-42 |
| **1.1 The manuscript also reports that the review follows PRISMA guidelines. All relevant elements of PRISMA were incorporated into ENTREQ with the caveat that ENTREQ guidelines do not explicitly state that the flow of literature should be reported with a PRISMA type diagram. Please clarify.** |  |
| Thank you for bringing this is our attention, this reference to the ‘PRISMA guidelines’ has been removed from the manuscript. | Pg. 2 |
| **2. It is more usual to consider 'the factors that create barriers and enablers to'...** |  |
| The authors agree, however, this is a direct representation of the question as it appears in the Priority Study (Healy *et al.,* 2018) and as such has not been revised. | Pg.2 |
| **3. The convention as you state is to report number of studies and number of articles. This also needs to be reflected in the flow diagram. The last box needs to reflect number of studies and number of articles.** |  |
| This inconsistency, which was also noted by Reviewer #2, has been corrected. | Fig.1 |
| **4. Please cross check reporting against the journal headings and subheadings for reporting reviews and syntheses. See for example your heading 'methodology of synthesis'. This is not a usual heading. 'Synthesis methodology' or just 'Synthesis' is more appropriate. I could not however find a 'data extraction/ abstraction subheading'. As the Thomas and Harden method combines data abstraction and synthesis you could see if a 'Data abstraction and synthesis heading' is agreeable to the journal.** |  |
| The manuscript heading has been amended to reflect this suggestion.  *Revised text:*  “Data abstraction and synthesis” | Pg. 15  Ln. 25 |
| **5. PROSPERO is becoming more flexible concerning the protocols they publish. Arguably recruitment rates to clinical trials or not is an important clinical indicator. Did you try registering the review or did you just assume that it did not meet their increasingly flexible criteria?** |  |
| The authors submitted a copy of the review protocol for registration with PROSPERO on 24/01/19. However, the submission was rejected on 26/03/19 based on the fact that the review did not have a primary direct patient or clinical outcome. This decision was queried by the authors on 12/04/19, citing examples of reviews with similar primary outcomes, unfortunately this too was unsuccessful in overturning the PROSPERO decision.  *Revised text:*  “The protocol for this QES, was considered by the team at PROSPERO as outside the scope of PROSPERO registration, as it does not address health outcomes. However, it is publicly available online (<https://osf.io/g4dt9/>).” | Pg. 6  Ln. 144-146 |
| **6. Some of the terminology used to describe included study types is a bit unconventional (such as visual and collaborative studies). Suggest stick to naming the types of types of qualitative methodologies and methods that would meet the inclusion criteria and be consistent throughout the paper.** |  |
| The authors agree, the unconventional terminology has now been removed.  *Revised test:*  “We included all qualitative studies, such as grounded theory studies, phenomenological studies, ethnographies, and case studies.” | Pg. 7  Ln. 154-155 |
| **7. Definition of a finding (p7) 'This review defined a finding as a statement made by a study author'. This feels too restrictive and is open to misinterpretation. A finding can be a theory, a new concept, a typology, a theme, subtheme, findings statement, impact statement etc etc, findings can be presented in tables, diagrams, infograms, pictures, summary statements additional online only files, abstracts, summary bullet points etc etc (ie anywhere in the paper). The suggestion is to rewrite 'what is a finding' and to articulate that findings can be found anywhere in the paper. A theoretical lens for framing findings for example can be found in the introduction or methods section of a primary study.** |  |
| The authors have revised the text in line with this suggestion and relocated the definition of ‘finding’ to under the heading “Data abstraction and synthesis” later in the manuscript.  *Revised text:*  “Findings were defined as any qualitative data describing a theory, new concept, theme, sub-theme or finding statement, presented in forms including, but not limited to, text, tables, diagrams, supplementary files located anywhere in the paper.” | Pg. 15  Ln. 35-37 |
| 8**. Anthropomorphism. Please avoid giving the review human qualities. The review is an object - it cannot do anything. See for example: 'This review defined...' Rewrite as 'Findings were defined as ........'** |  |
| This has now been rectified - see above comment | Pg. 15 |
| **9. Data extraction. With the Thomas and Harden's method of thematic synthesis, it is usual to code the entire paper and develop the synthesis using a software product. The text suggests that data were extracted into a table and then coded? It is more usual to extract the study information and characteristics into a table and then upload the entire manuscript and additional files to a software product for coding and synthesis. Please clarify.** |  |
| It was decided that as the QES was part of a body of work for a PhD, and that the number of papers included were very small, there was an opportunity to manually code data so that the author (VH) could develop her skills with engaging with and immersing herself in the data rather than also having to engage with learning the process of the software package We have clarified the manuscript to refer to this.  *Revised text:*  “Given the small number of papers, we used MS Excel to manage the data and opted to code and syntheses data manually.” | Pg.15  Ln.30-31 |
| **10. Synthesis. The analytical themes presented in the findings section appear to be close to the primary studies and do not yet seem to move substantially away to develop new patterns and understandings that were not apparent in single primary studies. This becomes more apparent when you look at the summary of themes and subthemes presented in the supplemental files. The question then arises are these descriptive level themes rather than analytical themes? You may need to be clearer that with 4 studies there appeared to be less opportunity to 'go beyond' the primary studies to analytical level. Given that you only had 4 studies, you may not have been able to advance to a truly analytical level without the input of extensive stakeholder consultation and building in a theory development stage in which you 'bridge the gaps' with new hypotheses. The other option would have been to see if any of the primary qualitative studies were linked to interventions (ie trial sibling studies) where you could have made better use of the data in it's original context to explain how a specific trial recruited and the potential impact of staff behaviours on recruitment rates. The key is not to overplay your findings if they really are not truly 'analytical' level. You may have a combination of descriptive with some reaching up into analytical - but at face value they don't look like stand alone analytical findings that move beyond the primary studies. Simply revising the theme head to something different but similar is not sufficient.**  **If not able to further advance the findings to a truly analytical level then suggest be transparent and outline the additional strategies that you could potentially have used in the limitations section. Please reconsider, and clarify.** |  |
| On reflection the authors agree that the findings are a combination of themes ranging from descriptive to reaching into an analytical level. The ability of the authors to ‘go beyond’ descriptive themes with the primary studies to a truly analytic level was somewhat limited due to small number of studies included in the review. A potential strategy to address this could be seeking extensive stakeholder input and including a theory development stage to ‘bridge the gaps' with new hypotheses. The manuscript has been amended in the ‘Strengths & limitations’ section to reflect the reviewer’s suggestions.  *Revised text:*  “The themes outlined in this review are a combination of descriptive and, at times, reaching into analytical level. Our ability to advance to a truly analytical level was somewhat curtailed by the number of studies (four) and therefore data available to the authors. A potential strategy to address this could be to seek extensive stakeholder input and the inclusion of a theory development stage to ‘bridge the gaps' with new hypotheses. Or alternatively, investigate if any of the primary studies were linked to interventions (i.e. trial sibling studies), granting the opportunity to make use of the data in its original context to explain how a specific trial recruited and the potential impact of staff behaviours on recruitment rates.” | P.36  Ln. 401-426 |
| 10.1 if able to make a more convincing case for analytical level findings, it would also be helpful for the reader to distinguish between descriptive level and analytical level themes in the text and the tables. |  |
| See above comment |  |
| **REVIEWER #2** |  |
| **Abstract conclusion refers to ‘combing’ – should this be ‘combining’?** |  |
| Thank you for highlighting this, the manuscript has been amended.  *Revised text:*  “theme combining” | Pg. 3  Ln. 61 |
| Methodology- some authors use thematic synthesis in a very broad sense. Suggest in the abstract you make reference to Thomas and Hardy’s approach specifically. |  |
| The text has been revised to include this suggestion.  *Revised text:*  “The synthesis was undertaken using Thomas and Harden’s three-stage thematic synthesis method and reported following ENTREQ guidelines.” | Pg. 2  Ln. 41-42 |
| **Table 1: please add the reference numbers for included papers otherwise readers need to scan the full reference list to find these.** |  |
| We have now included the reference numbers. | Pg. 11-13 |
| **Reflexivity: I was very pleased to see such information provided. I would suggest though that the information about the researchers is added to your strengths/limitations section on p36 as** **your professional background is the context in which your interpretations/analysis are made so, it helps readers to understand this when they are reading about the strengths/limitations of your study.** |  |
| Thank you very much for this suggestion, the authors agree and have now included reflexivity in the strengths and limitations section of the manuscript. | Pg. 37  Ln. 421-426 |
| **Findings: page 20. A) You note five papers from four studies but this does not match your PRISMA diagram which says five studies. Please amend your PRISMA diagram to say 4 studies, 5 papers. B) Could you report somewhere whether these 4 studies were found via the electronic searching or if any were found via the grey literature search? References suggest these were from the e-searches but, it is useful for readers to know this in terms of study quality. Perhaps add to page 20 all identified papers were found through database searching.** |  |
| This oversight has also been noted by Review #1. The manuscript amended accordingly.  A) PRISMA diagram amended  B) Details on where papers were found has now been added to the manuscript  Revised text:  “This synthesis of qualitative evidence includes five papers, from four studies, all of which were identified through an electronic database search.” | Fig. 1  Pg. 20  Ln.3-4 |
| **Strengths and limitations and confidence of findings: Perhaps these sections could be merged but with more detail added?** |  |
| Thank you for this suggestion and we agree there are many ways we could have presented this. The authors have made the decision to present the Confidence of Findings separately. We followed the format of a recently published QES in PLOS One and think this best captures the importance we place on the GRADE-CERQual assessment.  <https://doi.org/10.1371/journal.pone.0211829> | Pg. 30  Ln. 260-268 |
| **You raise key points but you need to go further. For example, your team consists of two midwives and a trial methodologist working in Scotland and Ireland. Team make up e.g. discipline, gender, is likely to have influenced your thematic synthesis. Perhaps a team of obstetricians may have interpreted the original study findings in a slightly different way to you as they would approach this synthesis from a different professional perspective? Please can you add something to this effect in this section of your paper – just the point that your final synthesis reflects your professional and personal backgrounds. Also, please comment that your included studies were from UK and Netherlands so findings reflect a European perspective from two countries with universal healthcare.** |  |
| *Revised text:*  “The studies included were from UK and Netherlands, therefore it should be noted these findings are reflective of a European perspective and are from two countries with universal healthcare.”  The authors appreciate these valuable suggestions. Undoubtably, our professional backgrounds are likely to have influenced our thinking and decision-making process, the manuscript has now been revised to include them.  “We acknowledge that the gender and make-up of the research team (two midwives and a trial methodologist) working in Ireland and Scotland is likely to have influenced thematic synthesis. Undoubtably, our interpretation of the primary studies and approach to synthesis is a reflection of our professional and personal backgrounds. However, we consider our reflexivity throughout the data analysis process, has strengthened the validity and rigor of the QES.” | Pg. 36  Ln.415-417  Pg.37  Ln. 422-426 |
| **Figure 2: I liked this but felt it was incomplete. A) The title needs improved it was too vague e.g. maybe it could mention themes? B) A point for consideration – could the 4 themes/columns be brought together in some way e.g. within a box? Perhaps providing an over-arching theme such as ‘recruiters’ perspectives of recruiting women during pregnancy and childbirth to clinical trials: 4 dimensions informing their practice’. At the moment, it’s simply a visual representation of your descriptive narrative. I feel there is potential for Fig 2 to say more – perhaps linking it to your interpretation/discussion so, it goes beyond just summarising your themes. This sort of thing could give your figure more impact and meaning to readers.** |  |
| Thank you for these helpful comments. The authors agree with the suggestions and have developed the diagram further to incorporate the ideas outlined above. | Pg.20  Fig 2 |
| **S3 – one column is missing its reference** |  |
| Thank you, this has now been amended. | S3 |
| **REVIWER #3** |  |
| **1. Suggest avoid ‘underpinned’ and replace with ‘linked’ or other rewording. Describing an ‘overarching theme’ as an ‘underpinning’ comes across as a contradiction.** |  |
| Thank you for this suggestion, this has now been amended in the manuscript.  *Revised text:*  “The overarching theme combining knowledge and power links the experiences and perceptions of recruiters.” | Pg. 3  Ln. 61-62 |
| **2. P4 Final sentence of first paragraph: Do the authors feel that findings could have relevance to trial recruiters in other clinical areas? This is mentioned on p34 second paragraph although not stated explicitly there or here. Perhaps something to comment on more explicitly either here or in the discussion.** |  |
| The authors are cautious when offering comments re the generalisability of the findings given that this QES sits within the space of maternity care. Pregnant women are not unwell and so recruitment to these trials can be different from other contexts. It may be that there are parallels with trials in paediatrics/neonatal care where parents as consenting on behalf of their child than other trials, but this would need to be explored.  See similar comment #18 |  |
| **3. P15 Methodology of synthesis: The authors have used the ENTREQ Checklist well in presenting this review. Whilst I accept that a thematic synthesis is justified for the study question, the authors have not yet clearly stated the rationale for conducting a QES/using thematic synthesis (as opposed to using other synthesis methodology). See Booth et al. 2016 [18] for clear arguments for why a thematic synthesis is appropriate for this study.** |  |
| The authors choice of method of synthesis was directed by criteria outlined in the RETREAT criteria (Booth 2018), this framework supported the selection of thematic synthesis as an appropriate method.  *Revised text:*  “Guided by the RETREAT framework(33), we undertook this qualitative evidence synthesis using a thematic synthesis method by Thomas & Harden(34). This method was chosen as it draws on the conceptualisation and techniques of thematic analysis in order to facilitate the transparent identification and development of descriptive and analytical themes. | Pg. 15  Ln. 27-30 |
| **4. Did the line by line coding reported here include all text in the findings and discussion of the papers reviewed – currently the authors refer to ‘all relevant text’ and it is not clear how a decision on relevance was reached. I was not initially clear whether it was simply participant quotes or also concepts proposed by authors in primary studies (first and second order concepts) and this only became clear on reading Table S5.** |  |
| The authors have revised and clarified these points in the manuscript.  *Revised text:*  “All qualitative findings from the primary studies relevant to the research question were extracted. Findings were defined as any qualitative data describing a theory, new concept, theme, sub-theme or finding statement, presented in forms including, but not limited to, text, tables, diagrams, supplementary files located anywhere in the paper.” | Pg. 15  Ln. 34-37 |
| **5. The authors have not yet made completely clear whether primary studies were analysed in a particular order or whether analysis was iterative across all study data.** |  |
| Papers were ordered alphabetically, and data extracted in order then a more iterative process was applied, going back and forth between papers during synthesis.  *Revised text:*  “Papers were ordered alphabetically, and data extracted in order.”  “Through a line-by-line coding process (VH) we developed ‘free codes’ (without hierarchical structure), this bank of codes grew as each paper was coded. An iterative process was then applied as data was examined and checked (VH, LB) for meaning and content, this process allowed the translation of codes and concepts between studies. As similarities and differences between codes became apparent, we grouped them into a hierarchical structure to form descriptive themes (VH, LB). Finally, we attempted to generate analytical themes from each category to go beyond the ﬁndings of the original studies into a higher order abstraction of the phenomena (VH, LB, KG) (see S3 Table).” | Pg. 15  Ln. 33-34  Pg. 15  Ln.38-45 |
| **6. P20 sixteen summary of qualitative findings statements are reported (Table 2) but I was not clear from the analysis how these were derived – were they derived after the themes were identified or were they derived then grouped together to form themes?** |  |
| The Summary of Qualitative Findings were derived from the findings of the thematic synthesis, the authors then made an overall GRADE-CERQual assessment of confidence based on these findings.  *Revised text:*  “A ‘Summary of Qualitative Findings’ were derived from the findings of the thematic synthesis, as the research team summarised and identified the central idea of each finding, the authors then made an overall GRADE-CERQual assessment of confidence on these findings. “ | Pg. 16  Ln. 56-59 |
| **7. P17-19 Table 2: Some content assigned to different themes appeared very closely related, so I was not clear how authors arrived at these themes (see above). Please see summary of qualitative findings statements given in the table below for my alternative interpretation of these SQF statements into three themes (1. Clinician first, recruiter second, 2. Recruiter beliefs about trial, participation and women, 3. Impact of context). Apologies if I have misunderstood the analysis process and I accept the authors are more familiar with the raw data, which may account for the themes presented.** |  |
| Thank you for these suggestions of an alternative interpretation of the SQF statements. The authors agree that there are areas overlap between themes, some content assigned to different themes is closely related but distinct. Through team discussion and consensus, the authors made judgement calls, arguably different teams may come up with different interpretations. This point has now been addressed in the ‘Strengths and Limitations’ section of the manuscript. | Pg. 17-19 |
| **8. General point re findings: I was not always clear which were the authors’ interpretations of the data, which were the interpretations of the primary study authors and what were those of the original research participants – although this became clearer reading S5 Table. Is it possible to signpost this in the text? (p8 France et al. 2019 i.e. distinction between first-order constructs, second order constructs and third order constructs: https://bmcmedresmethodol.biomedcentral.com/articles/10.1186/s12874-019-0670-7 ).** |  |
| Thank you for highlighting the paper by France *et al.,* The authors note that this paper refers to a meta-ethnography and as we are using Thomas & Harden’s thematic synthesis it would not be appropriate to begin to introduce the labelling of 1^st^, 2^nd^, 3^rd^ order constructs.  For ease of the reader we have chosen not to include data from the included studies (whether that be in the form of participant quotes or authors interpretations), however this information is provided as a supplementary table (S5) which we have now colour coded to visually distinguish between primary study participant quotes and study author interpretations. | Pg. 20-30 |
| **9. P20-21 There is no mention in the text whether views and experiences differed across these professional groups – I was particularly interested to know if there was variation across professions in views expressed under 1.2: ‘These judgements were based on their perceptions that pregnant women lacked the capacity to understand and make decisions around trial information.’ I see from S5 Table that these views are held across professions I was also curious to know whether any patterns of assumption emerged according to professional group but numbers may have been too small.** |  |
| While it would have been interesting to explore the variation of experiences across different professional groups, unfortunately the number of participants in the primary studies was insufficient to perform subgroup analysis in the context of this QES. However, the origin of the direct quotes which is provided in table S5. | Pg. 20  Ln. 18-19  (S5) |
| **10. The authors mention that midwives were regarded by fellow professionals as gatekeepers – was the use of paternalistic language e.g. ‘our girls’, ‘my ladies’ limited to midwives or common across all professions?** |  |
| This is an interesting inquiry, and one the authors cannot definitively answer as they can only interpret the data that is presented in the primary studies. In this instance, the review was only able to identify quotes from midwives using paternalistic language as presented in the original studies (see S5). | Pg. 24 |
| **11. P22 suggest ‘receptive’ to replace ‘receptable’** |  |
| Noted | Pg. 22 |
| **12. P22 suggest ‘implied’ in place of ‘inferred’.** |  |
| *Revised text:*  “…language that implied…” | Pg. 22  Ln. 71 |
| **13. P31 The authors sum up their key findings as follows, arguing they have discovered an:**  **‘overarching theme which combines knowledge and power. In other words, our review suggests that when it comes trials recruitment, recruiters believe they know ‘best’ Recruiters often cite their assumed knowledge and wisdom regarding women’s preference, capabilities and personal circumstances alongside their judgements and assumptions in relation to the trial and the trial intervention’. (p31, first paragraph of discussion).**  **I agree with the suggestion that there is an interaction between (a lack of) knowledge (or perhaps beliefs rather than knowledge) and power, but I wonder whether this summary of the findings does not make this interaction explicit enough? Is perhaps the key issue that recruiters believe they know best from a clinicians’ perspective (when from a trial conduct perspective they may not) and may act in line with their (clinical) beliefs rather than in line with trial protocol, e.g. in exercising their beliefs about which individuals they should approach rather than approaching all eligible women (gatekeeping which has implications for recruitment and potentially generalisability of findings). If that is the authors’ understanding, I would like to see this argued clearly here. This then also supports the argument for the need to ensure recruiter training (Implications for practice p 34) as these beliefs may be addressed in training that increases recruiter understanding of trial conduct.** |  |
| Thank you for this very useful point of feedback. On reflection we can see why this point has been raised. We had identified the overarching theme of ‘knowledge’ based on our understanding of the concept of authoritative knowledge (Jordon 1992), when one kind of knowledge gains ascendance and legitimacy and consequently other kinds are dismissed. However, on reflection of this comment we can now appreciate that the use of the label knowledge may be confusing and so cloud our meaning. We therefore welcome your suggestion of relabelling the overarching themes to ‘beliefs and power’.  *Revised text:*  “Our discussion considers the most salient findings threaded throughout the synthesis of evidence, linked by an overarching theme which combines beliefs and power. In other words, our review suggests that when it comes to trial recruitment, recruiters believe they know ‘best’.” | Pg. 31  Ln. 274-277 |
| **14. P31 second paragraph in the Discussion – I was not clear on the key point that is made and illustrated here. Perhaps that recruiters make assumptions that are often over negative which results in people being excluded from trial participation? Important to make a reference to the implications of this for the generalisability of trial findings (referenced in introduction but helpful to reference here). Maybe the authors could make more explicit that** **the concept of vulnerability for pregnant women is coming under challenge in the literature but this may not have filtered through to clinical practice.** |  |
| The authors have amended the manuscript to clarify the points raised.  *Revised text:*  “Our synthesis shows recruiters frequently adopt a position of thinking they know ‘best’ in making judgements and assumptions, which were often overly negative which resulted in women being excluded from trial participation.…  …The implications of restricting trial access has potentially far reaching consequences for the health of pregnant women and their unborn babies, and limits the ability to make evidence-based clinical decisions(13). Several recruiters referred to pregnant women as vulnerable and deserving of protection from the demands of trial involvement. This finding is commonly reported in the literature (37, 38), and may provide an important insight in understanding the cause of the underrepresentation of pregnant women in clinical research (9). More recently, the concept of vulnerability in pregnancy is coming under challenge in the literature (11), suggesting that pregnancy should not be considered a deterrent for inclusion in clinical research, however, this concept may not have filtered through to clinical practice.” | Pg. 31  282-295 |
| **15. P32 Has the paternalistic language used been reported and critiqued elsewhere in the literature given how striking its use is in this setting? I see the reference to a blog from 2018 but I am curious to know of any other critiques.** |  |
| The authors have sought further literature to support this finding and amended the manuscript accordingly.  *Additional reference:*  Furber & Thomson 2010 | Pg.32  Ln. 305 |
| **16. P33 Paragraph beginning ‘The overarching concept of knowledge’ – perhaps add a comment here that** **there was evidence of recruiters acting ‘with confidence’ but in a way that contradicted trial protocol, i.e. in line with recruiter beliefs rather than inline with deeper understanding of trial aims and processes.** |  |
| Thank you for highlighting this, the authors have included this suggestion in the manuscript.  *Revised text:*  “The overarching theme of beliefs is threaded through the ‘doing’ of recruitment, which was evidenced by the way recruiters acted with confidence, aligning their approach to recruitment with their own beliefs which often contradicted trial protocol.” | Pg. 34  Ln. 335-337 |
| 17. P34 re ‘variation in the approach to the recruitment encounter’. Might this variation be necessary and justified given the wide range of trial designs, point on pathway and setting where recruitment can occur? |  |
| Yes, indeed, and this is why a ‘one size’ approach to recruitment may not be appropriate.  *Revised text:*  “The considerable variation in the approach to the recruitment encounter, ranging from team involvement to timing and exchanging trial information, is justifiable given the wide range of trial designs, point on pathway and setting where trial recruitment during pregnancy can occur.” | Pg. 34 Ln. 346-349 |
| **18. P35 the authors argue findings could have relevance to other key priorities of research identified by the Priority Study. Maybe also comment on the generalisability of findings for recruiting in other clinical specialties. This is touched upon on p34, first full paragraph but could be brought out more.** |  |
| The question of generalisability is similar to comment #2. The authors are cautious in their approach to stating the generalisability of the findings outside recruitment in the maternity care setting. |  |
| **19. P35 First sentence under Implications should read ‘Interventions such as the QuinteT Recruitment Intervention…** |  |
| The manuscript has now been amended.  *Revised text:*  “Interventions such as QuinteT Recruitment Intervention” | Pg. 35  383 |
| **20. P36 Felt this summed up key findings really nicely 1) interaction between belief and power 2) gap between study design and lived reality and how to address these by involving recruiters as key stakeholders at the design phase and providing ongoing training during trial delivery.** |  |
| The authors appreciate your commendation of the conclusion. |  |
| **Some variation in tense used – occasional switch from past to present. Helpful for reader to keep consistent to past throughout.** |  |
| Thank you, this has been noted. |  |
| **Please check use of semi-colon vs colon.** |  |
| Thank you, this has been noted. |  |
| **P22 Sentence beginning ‘Whilst recruiters in Stuart’s and Lawton’s studies … please review sentence construction** |  |
| The sentence has been amended.  *Revised text:*  “Furthermore, in two of the studies recruiters expressed the view that potential trial participants did not have the ability to read or were illiterate” | Pg.22  Ln. 61-62 |
| **Check for use of ‘inferred’ to mean ‘implied’** |  |
| Noted, thank you for highlighting this.  *Revised text:*  “…language that implied…” | Pg. 22  Ln. 71 |
| **P32, first full paragraph, second sentence beginning ‘Particularly striking…’ please check grammar, phrasing.** |  |
| *Revised text:*  “The expressions of professional ownership, such as ‘my girls’ are particularly striking…” | Pg.32  303-304 |
| **P32 First sentence of second paragraph: perhaps ‘illustrated in the language of paternalism’?** |  |
| *Revised text:*  “illustrated in the language of paternalism” | Pg.32  Ln. 302-303 |
| **S2 Columns 5 and 7 need resizing to show all text.** |  |
| This has now been amended. | S2 |
| **Should the entry for Stuart et al. by LB in column 8 be entered in column 10? (4. Is data analysis described and is this appropriate – comment)** |  |
| This has now been amended. | S2 |
| **Given that none of these studies were judged to** **include evidence of reflexivity is this worth drawing attention to in the discussion, as there is clearly a need to improve this aspect of in future studies?** |  |
| This is an important issue and we have now included it in the ‘Implications for future research’ section.  *Revised text:*  “Furthermore, there is a clear need for future primary qualitative research to include evidence of reflexivity, to provide context to, and a deeper level of understanding of the interpretations and analysis made.” | Pg. 36  Ln. 397-399 |
| **Possible organisation of SQFs – Table example** |  |
| The authors have responded to this point in comment #7 above.  The table of Summary of Qualitative Findings (Table 2) is presented in line with a GRADE-CERQual specific template for reporting (Lewin *et al.,* 2018) [*https://implementationscience.biomedcentral.com/articles/10.1186/s13012-017-0689-2*](https://implementationscience.biomedcentral.com/articles/10.1186/s13012-017-0689-2)  *Revised text:*  “Table format from Lewin *et al.,* 2018 (36)” | Pg. 17-19 |
